# Revolutionising healthcare access: student-led clinics as catalysts for change in an underserved urban community in Karachi, Pakistan

**DOI:** 10.7189/jogh.16.04262

**Published:** 2026-09-11

**Authors:** Syed Muhammad Aqeel Abidi, Ali Hyder Nazeer, Bilal Lodhi, Syeda Kainat Fatima, Fatima Abdullah, Muhammad Ali Akbar Khan, Maheen Zakaria, Ali Azan Ahmed, Aly Hamza Khowaja

**Affiliations:** 1 Medical College, Aga Khan University, Karachi, Pakistan; 2 Humanity Initiative NGO, Karachi, Pakistan

**Keywords:** student-led clinics, universal health coverage, health literacy, primary healthcare, underserved populations, low- and middle-income countries

## Abstract

**Background:**

Karachi, Pakistan’s largest metropolitan city, faces profound healthcare disparities, with an estimated 45–49% of the population residing in underserved informal settlements. Within these communities, the primary healthcare system is not merely under-resourced but structurally fractured, leaving patients to navigate prohibitively expensive private providers or overcrowded public hospitals. To address these systemic gaps, the Humanity Initiative, a student-led non-governmental organisation, implemented student-led clinics (SLCs) as a community-embedded, cost-effective model to bridge critical care deficits.

**Methods:**

We conducted a descriptive study examining the implementation and early outcomes of two SLCs conducted in Agra Taj Colony, Lyari Town, Karachi. Operating every three weeks under licensed physician supervision, each SLC provided free primary consultations, dispensed essential medications, and delivered structured health education. We analysed patient demographics, disease burden, operational costs, and process-level indicators across both clinic cycles.

**Results:**

A total of 371 patients were seen across SLC1 (n = 194) and SLC3 (n = 177), with a higher proportion of female patients (n = 224, 60.4%). The disease burden was dominated by non-communicable diseases, including hypertension (14.3%) and diabetes (8.6%), alongside a notable number of acute infections and multi-system complaints reflecting the dual epidemiological burden characteristic of urban low- and middle-income country (LMIC) settings. Operational costs were maintained at PKR 128–136 (USD 0.45–0.48) per patient, demonstrating exceptional cost-efficiency. We found significant gaps in health literacy, particularly regarding chronic disease self-management, and gendered barriers to healthcare access.

**Conclusion:**

The SLC model demonstrates proof-of-concept as a viable, scalable strategy for bridging structural gaps in fractured primary healthcare systems in LMICs. By embedding care within the community, SLCs advance Universal Health Coverage, rebuild community trust in allopathic medicine, and provide supervised experiential learning opportunities for future healthcare professionals. Replication of this model across similar underserved communities holds significant potential for reducing healthcare inequity in resource-constrained urban settings.

Karachi, a mega-city of over 20 million residents, serves as a microcosm of the healthcare challenges confronting low- and middle-income countries (LMICs). Despite its status as Pakistan’s foremost economic hub, nearly half of its population resides in *katchi abadis* (*i.e.* informal urban settlements) characterised by severe overcrowding, inadequate sanitation, and a near-total absence of formal healthcare infrastructure [1]. In these settings, the theoretical ‘hub-and-spoke’ primary healthcare model, linking community outreach workers to local clinics and onward to tertiary facilities, exists largely on paper. In practice, the connective tissue between these nodes has broken down: patients are left to navigate a labyrinth of expensive private providers or overcrowded public hospitals, frequently resulting in delayed presentations, inappropriate self-medication, and catastrophic out-of-pocket expenditures [2–4].

Beyond financial and geographic barriers, two less-visible but equally formidable obstacles stand between these communities and formal care: deficits in health literacy and a profound erosion of trust in allopathic medicine [3]. In the absence of accessible formal providers, many residents rely on informal dispensers, pharmacists, or traditional healers, not merely due to cost, but because the formal health system feels administratively intimidating, distant, and inaccessible [5,6]. These dynamics perpetuate a cycle of late-stage presentations and poor chronic disease control that disproportionately burdens the most vulnerable [7,8]. With Karachi’s population projected to continue expanding, these structural gaps threaten to widen unless addressed through contextually adaptive, community-embedded solutions.

Student-run free clinics (SRFCs) have a well-documented history in high-income countries (HICs), originating in the social activism of 1960s USA and subsequently expanding across North America, Europe, and Australia as tools for both health service delivery and medical education [9,10]. As of 2014, the society of SRFCs had catalogued over 200 affiliated clinics operating across multiple countries [9]. In HICs, SRFCs function as safety nets for the uninsured; in Pakistan and similar LMICs, the challenge (and the opportunity) is fundamentally different. We propose that student-led clinics (SLCs) must function not merely as safety nets, but as structural bridges that reconnect severed spokes within fractured primary healthcare systems.

To address these structural gaps, the Humanity Initiative (HI), a student-led non-governmental organisation (NGO), developed and implemented SLCs in underserved urban communities. These clinics provide free primary consultations, essential medications, and preventive health education under the continuous supervision of licensed physicians [11]. The SLC model actively engages medical students in direct patient care, facilitating both a tangible expansion of healthcare access and the development of clinical and professional competencies in the next generation of practitioners [12,13]. Unlike conventional SRFCs operating within structured, well-resourced health systems, the HI model is explicitly designed for resource-limited, fragmented care environments, representing a meaningful contribution to the limited literature on SRFC implementation in LMICs.

Grounded in the World Health Organization’s Universal Health Coverage framework and Sustainable Development Goals (*i.e.* Good Health and Well-Being), we present the HI SLC model as a proof of concept for community-embedded primary care in urban LMICs. We document operational design and cost-effectiveness data, and aim to provide a replicable blueprint for interventions to rebuild community trust, advance health literacy, and strengthen care systems.

Our primary objective is to document, describe, and evaluate the HI SLC model for primary healthcare delivery in an urban LMIC setting. Specifically, we aim to: demonstrate the operational feasibility and cost-effectiveness of the model; characterise the demographic profile and disease burden of the target community; establish a transparent, replicable implementation blueprint for similar initiatives; and identify operational challenges and lessons learned to inform future scale-up and adaptation.

## METHODS

### Site selection and community partnership

We conducted this study in Agra Taj Colony, Lyari Town, a historically underserved and socioeconomically deprived urban neighbourhood. Site selection was guided by four criteria. First, community need, evidenced by severely limited primary care access, high poverty prevalence, and inadequate healthcare infrastructure. Second, physical infrastructure: we established a partnership with the Al-Qaim Trust (AQT), a well-rooted local organisation that provided access to an existing community health facility. On non-SLC days, this facility hosts two physicians (including a paediatrician), providing a foundation of clinical credibility within the community. Third, proximity to referral pathways: Lyari General Hospital, a tertiary care facility, is located approximately 2 km from the site, enabling timely onward referral where indicated. Fourth, community embeddedness: the AQT’s existing relationships with locals were essential for overcoming the initial hesitancy toward formal medical care that is characteristic of such communities.

This strategic collaboration was bidirectional: the SLC provided a consistent influx of trained medical volunteers and standardised clinical protocols to the facility, while AQT provided physical space, on-ground logistical support, and pre-established community networks. This community-partnership model is a defining feature of the HI approach and is consistent with evidence that embedding health interventions within existing community structures significantly improves uptake and sustainability [14].

Every three weeks, the SLCs provide consistent and predictable access to care within the community. We reported data from two clinic cycles: SLC1 (24 February 2023) and SLC3 (2 April 2023). Significant civil unrest and nationwide protests that disrupted access affected SLC2 (19 March 2023). Therefore, we did not include SLC2 data in this analysis.

### Volunteer recruitment and competency framework

Medical students organising and staffing SLCs are required to have completed at least one clinical rotation in an approved discipline before participation. Eligible rotations include internal medicine, general surgery, family medicine, otolaryngology, ophthalmology, paediatrics, and obstetrics and gynaecology. This prerequisite ensures that student volunteers possess baseline clinical exposure before engaging with patients. A mandatory pre-camp briefing is conducted for all volunteers.

Within each SLC, students are assigned roles matching their level of training. Junior volunteers (typically third-year clinical students) conduct structured history-taking, measure vital signs, and perform anthropometric assessments. Senior volunteers (in their penultimate or final year of clinical rotations, or recent graduates) undertake clinical examinations and contribute to initial management planning. Critically, licensed physicians, who maintain supervisory oversight throughout, make all diagnoses, treatment prescriptions, and referral decisions. This task-sharing structure aligns with World Health Organization-endorsed strategies for maximising human resources for health in resource-constrained settings and provides students with a scaffolded, real-world clinical experience that, particularly for students from private medical universities, may represent their only sustained exposure to community-based primary care.

### Patient flow and operational framework

**Figure 1:**
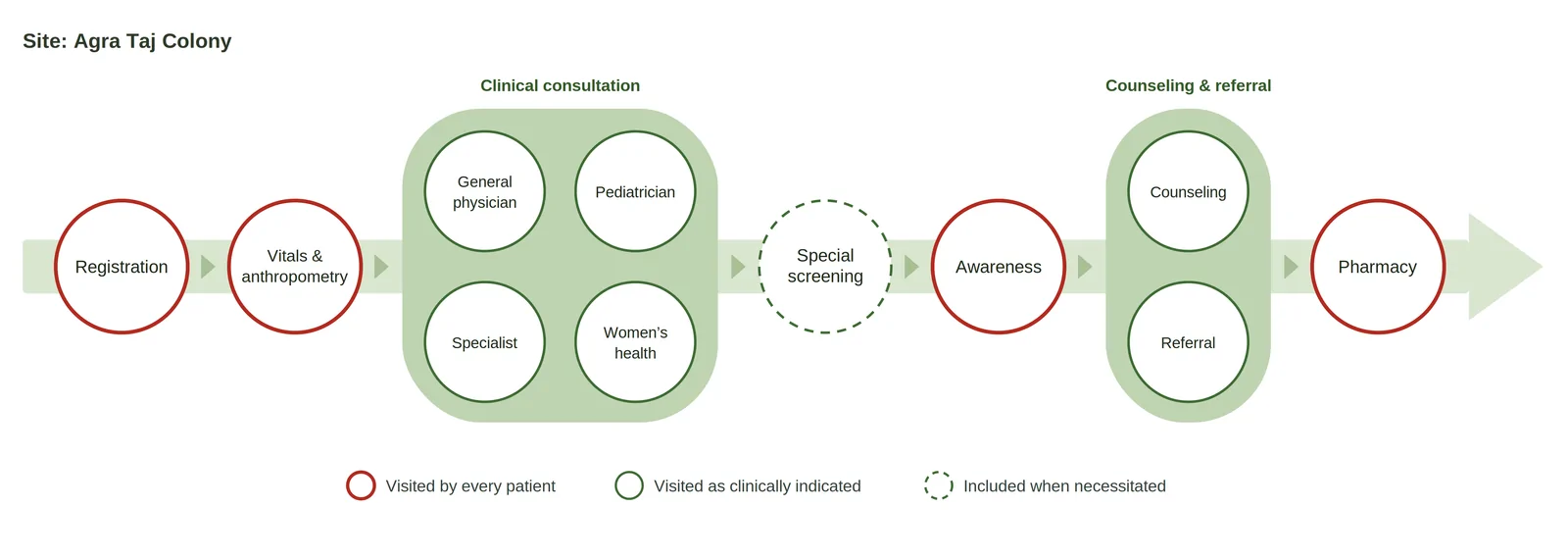
Diagram mapping out the flow of an SLC. Stations outlined in red are those which every patient must visit; a special screening station is part of the flow when necessitated. SLC – student-led clinic.

**Figure 2:**
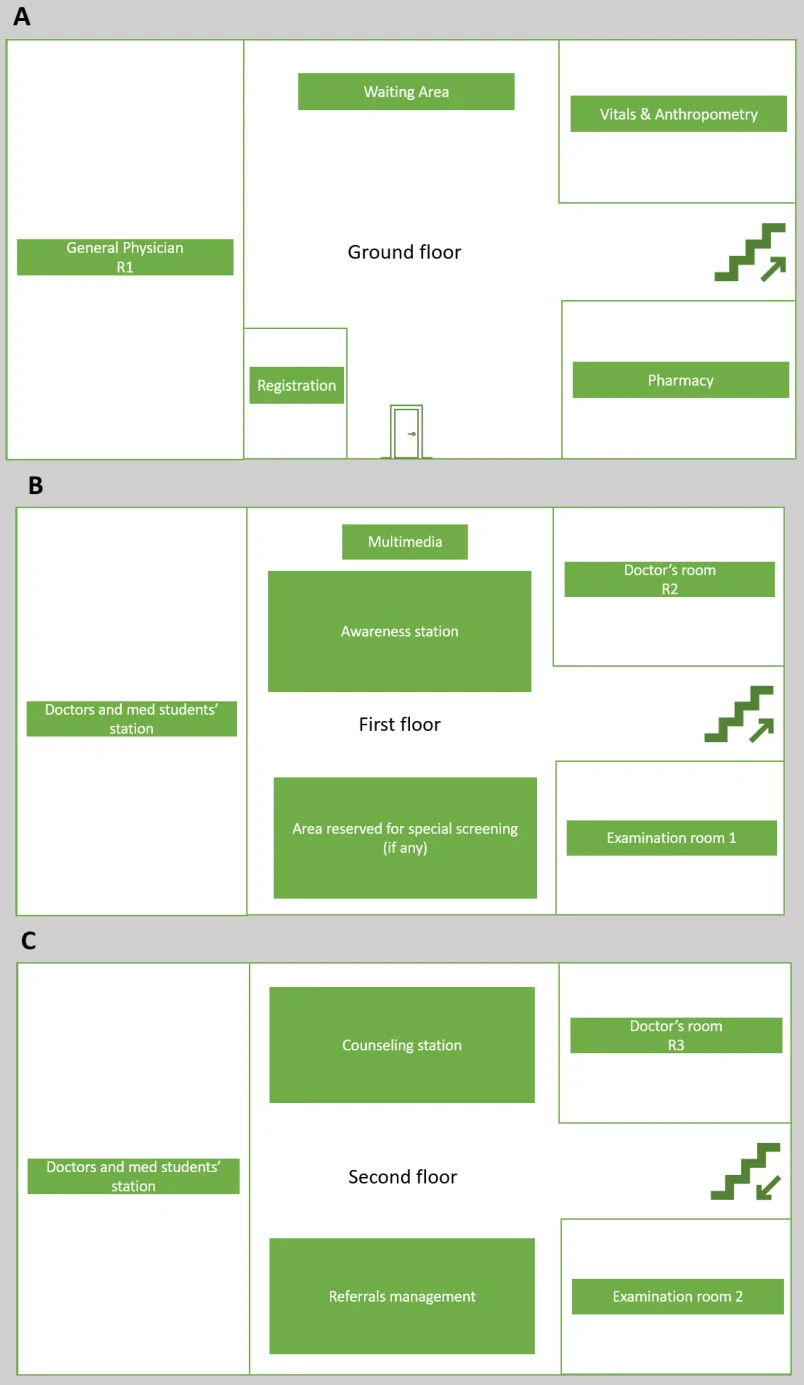
Floor maps of SLC. **Panel A.** Ground floor. **Panel B.** First floor. **Panel C.** Second floor. SLC – student-led clinic.

The SLC operates across three physical levels of the AQT facility using a structured, sequential patient flow (**Figure 1**; **Figure 2**, Panels A–C). Upon arrival, patients are registered at the ground-floor reception, where demographic details (name, age, contact number, and computerised national identity card (CNIC) number) are recorded and a unique encounter number is assigned. Returning patients are identified through cross-referencing with the existing directory. All patients subsequently proceed to a standardised vitals and anthropometry station, where blood pressure, oxygen saturation, respiratory rate, and age-appropriate anthropometric parameters are recorded on a standardised paper-based history form.

A brief triage inquiry on the presenting complaint, combined with age and mobility status, directs patients to one of three consultation rooms: the ground floor for elderly or mobility-limited patients seen by the general physician; the first floor for pediatric and women’s health cases; and the second floor for specialist consultations. Where required, specialists and volunteers descend to the ground floor for mobility-limited patients. On the first floor, an awareness station delivers structured group health education in Urdu or a relevant local language, addressing preventive care, chronic disease self-management, and health-promoting behaviours. A dedicated counselling station on the second floor is available for patients referred by the supervising physician. Patients requiring external investigations or specialist care are processed at the referral management station, where logistics and transport are coordinated under NGO sponsorship [15]. The encounter concludes at the ground-floor pharmacy, where medications are dispensed free of charge, and the completed history form is collected for digitisation; hence, every patient must visit the pharmacy regardless of whether any medications were prescribed.

### Community engagement and outreach

Community engagement is facilitated through designated focal persons embedded within the local population. Information about upcoming clinics is disseminated *via* WhatsApp broadcasts, community notice boards, and word-of-mouth networks. This combination of digital and interpersonal outreach has proven effective in driving consistent patient attendance.

### Data collection, management, and confidentiality

Data collected at each encounter includes demographic details (gender, age, marital status, residence, CNIC, and contact number); current comorbidities and medications; a structured medical history including presenting complaint and examination findings; provisional diagnosis; and treatment provided and disposition. The CNIC number functions as a unique patient identifier. For patients without a CNIC (including refugees and undocumented individuals), a contact number serves as an alternative identifier. Patient follow-up is managed by cross-referencing these identifiers against the clinic database.

A research and development team initially record the data on printed forms during the encounter and then transfer it to a secure database *via* Google Forms. Hard copies are retained for seven years before secure disposal; digital records are stored indefinitely in an encrypted, access-restricted database; and CNIC data are permanently deleted after five years. All procedures comply with the ethical guidelines of the HI NGO (Code of Good Research Practice).

## RESULTS

### Patient demographics and disease burden

A total of 371 patients were seen across two SLC cycles: 194 patients at SLC1 (24 February 2023) and 177 patients at SLC3 (2 April 2023). The patient population was predominantly female (60.4%) across both clinics. Most patients were middle-aged to elderly, consistent with the chronic disease burden (**Table 1**).

**Table 1:** Demographic characteristics of patients visiting both SLC camps (n = 371)

	n (%)
**Gender**	
Female	224 (60.4)
Male	147 (39.6)
**Age group in years**	
<18	71 (19.1)
18–40	43 (11.6)
41–60	136 (36.7)
>60	121 (32.6)
**Comorbidity status**	
Hypertension	53 (14.3)
Type II diabetes	32 (8.6)
Any comorbidities*	121 (32.6)

SLC – student-led clinic

*Any comorbidities category includes but is not limited to: hypertension, diabetes, ischaemic heart disease, chronic respiratory disease, autoimmune conditions, *etc*.

The disease profile across both SLCs reflected the dual epidemiological burden characteristic of urban LMIC communities: a high prevalence of non-communicable diseases (NCDs) coexisting with an active burden of communicable and acute illness. Among the most prevalent diagnoses, hypertension was the most common condition (14.3%), followed by type 2 diabetes mellitus (8.6%). Many patients with these chronic conditions, despite being aware of their diagnoses, demonstrated inconsistent medication adherence and significant gaps in understanding of long-term self-management, reinforcing the need for structured chronic disease education integrated into the SLC model.

Beyond NCDs, both clinics managed a range of acute complaints, including febrile illness, upper and lower respiratory tract infections, gastroenteritis, dermatological conditions, dizziness, musculoskeletal pain, and genitourinary infections. A subset of patients was identified with conditions requiring specialised assessment, including suspected hepatitis C and severe or complex infections; these were referred to partnered or subsidised tertiary facilities, with referral logistics coordinated by the NGO.

### Operational cost analysis

The cost-effectiveness of the SLC model is illustrated by the per-patient operational expenditure calculated across both clinic cycles (**Table 2**). In SLC1, total direct costs amounted to PKR 33,891 (**Table 3**). Dividing total costs by patients seen (n = 194) yields a per-patient cost of PKR 174.70 (USD 0.62). In SLC3, total direct costs were PKR 19,573, yielding a per-patient cost of PKR 110.58 (USD 0.39). The lower cost in SLC3 was because supplies were carried over from SLC1.

**Table 2:** Cost per patient summary across both SLC cycles

			Cost per patient*	
	**Date**	**Patients seen**	**PKR**	**USD**
**SLC1**	24 February 2023	194	174.70	0.62
**SLC3**	2 April 2023	177	110.58	0.39

SLC – student-led clinic

*USD conversion at the prevailing rate at the time of the clinic, taken as PKR 283.79 per USD 1 for all calculations in this manuscript.

**Table 3:** Itemised cost breakdown by clinic

	SLC1 (in PKR)	SLC3 (in PKR)
**Expenditure item**		
Medications	25,891	12,623
Equipment (glucometer, pulse oximeters)	7,100	6,650
Malaria rapid diagnostic kits (×10)	900	
Medication transport		300
**Total direct costs**	33,891	19,573
Estimated comprehensive cost per patient (including transport, printing)	180–200*	180–200*

SLC – student-led clinic

*PKR 180–200 equals approximately USD 0.63–0.70.

When additional indirect costs are incorporated, including volunteer transportation and printing materials, which were not included in the primary cost-per-patient calculation, the estimated comprehensive cost rises to approximately PKR 180–200 (USD 0.63–0.70) per patient. Transportation costs vary substantially across clinic sites, depending on the distance to the community and the number of volunteers mobilised for a given session. Despite this variability, even the upper-bound comprehensive estimate of USD 0.70 per patient is very low relative to the average cost of a private primary care consultation in Karachi, which typically ranges from PKR 500–1500 (USD 1.80–5.36), placing the SLC model at a fraction of prevailing market rates. For context, Pakistan's current health expenditure *per capita* stands at approximately USD 152 (purchasing power parity-adjusted, 2023 World Bank data), meaning the SLC model delivers a complete primary care encounter at roughly 0.4% of the national per-capita health expenditure [16].

## DISCUSSION

We present the implementation and early outcomes of SLCs as a proof-of-concept for community-embedded primary care. Across two clinic cycles, 371 patients received free primary care, with per-patient operational costs roughly 0.4% of the national per-capita health expenditure. Health literacy gaps, specifically around chronic disease self-management, emerged as a consistent and clinically significant finding, reinforcing the need for structured health education as an integral, rather than supplementary, component of the SLC model.

### Bridging a fractured system: SLCs as a structural intervention

The conceptual contribution of our study extends beyond the operational data. Pakistan’s primary healthcare system nominally follows a hub-and-spoke architecture, where community health workers and basic health units feed into district hospitals and ultimately tertiary facilities. In practice, however, the connecting pathways between these nodes are consistently dysfunctional [17]. Community workers are understaffed and under-supported, basic health units are frequently non-operational or inaccessible, and patients from communities like Lyari encounter a dead end where spokes should be [18]. The result is a pattern of delayed presentations, catastrophic out-of-pocket expenditure, and an erosion of trust in formal medicine that compounds access barriers over time [2,3].

The SLC model addresses this structural failure by providing a functional, trusted, and community-embedded node rather than rebuilding the formal system, which is beyond the scope of a student-led initiative. By operating from a familiar physical space, in partnership with an established local organisation and in a culturally concordant manner, the SLC effectively serves as multiple reconnected steps simultaneously. It provides primary care that would otherwise require a visit to an overcrowded public facility, offers onward referral pathways with logistic support, and delivers preventive education that strengthens the community’s own capacity for health self-management. Over the course of repeated, predictable visits, this consistent presence begins to rebuild the trust in formal medicine that fractured systems have eroded.

### Health literacy, referral pathways, and the role of structured education

A recurring observation across the SLC cycles was the gap between diagnostic awareness and self-management capability. Many patients with hypertension and diabetes were aware of their diagnoses but demonstrated inconsistent adherence to prescribed medications, limited understanding of dietary modifications, and a poor understanding of the consequences of uncontrolled disease. This pattern is not unique to Lyari: it reflects a well-documented gap in LMIC primary care settings between the passive transmission of a diagnosis and the active cultivation of health literacy [19]. The SLC model directly targets this gap through structured health education delivered at the awareness station, where sessions cover hand hygiene, vaccination schedules, chronic disease self-management, and pregnancy care, all delivered in Urdu or the preferred local language [20].

The referral pathway component of the SLC deserves particular emphasis because it addresses a major gap in informal care in communities like AQT: the lack of a functional referral route when a patient’s condition exceeds what community resources can manage [21]. By providing NGO-sponsored logistical support and actively tracking referrals, the SLC not only identifies conditions requiring specialist care (*e.g.* hepatitis C, severe infections, complex NCDs) but also helps ensure that patients complete the referral rather than abandoning care because of financial or logistical barriers. This referral tracking function transforms the SLC from a one-time episodic intervention into a longitudinal care management mechanism.

### Female engagement and gendered barriers to healthcare access

The higher proportion of female patients (60.4%) across both clinics is an epidemiologically and socially meaningful finding that warrants careful interpretation. In underserved urban communities in Pakistan, women face a compounding set of barriers to formal healthcare access: financial dependence that limits autonomous healthcare spending, mobility restrictions that make travel to distant facilities difficult, cultural norms that prioritise male family members’ health needs, and discomfort with male providers in the formal sector [22–24]. The higher female uptake at SLCs suggests that the community-embedded model, by removing geographic, financial, and cultural barriers simultaneously, may disproportionately benefit women who would otherwise go without formal care. Ensuring that female-specific services (*i.e.* women’s health consultations, obstetric referrals, genitourinary care) are consistently available at each SLC, and that female physicians and senior female students are recruited as volunteers, should be treated as a non-negotiable design principle for future cycles.

### Novelty in a low-resource context

While SRFCs are well-established in HICs, their implementation in LMICs remains limited and poorly documented in peer-reviewed literature. The HI SLC model differs from its HIC counterparts in three structurally important ways. First, it operates in an environment where diagnostic resources are severely constrained, where there are no on-site laboratory tests or imaging, and clinical decision-making mostly relies on history and examination, supplemented by a small number of point-of-care tests (*i.e.* glucometry, pulse oximetry, malaria rapid diagnostic tests). This demands a different kind of clinical reasoning from both students and supervising physicians, emphasising syndromic management, clinical pattern recognition, and careful risk stratification for referral. Second, referral pathways are constructed and managed by the SLC itself, rather than relying on pre-existing institutional pathways, a critical difference from HIC models where onward care systems are robust. Third, the model is dependent on community partnership for legitimacy and uptake. Without the pre-existing relationships of AQT, patient engagement would have been substantially lower, regardless of the quality of care offered [25,26].

The model also provides sustained, supervised community healthcare exposure, which conventional medical education in Pakistan’s private university sector rarely offers. For some students, participation in SLCs may represent the only opportunity during their undergraduate training to practice clinical medicine in a resource-limited community setting, encountering the full complexity of social determinants of health, diagnostic uncertainty without investigative support, and the human dimensions of healthcare access. This experiential dimension of the model aligns with global calls for medical curricula that cultivate both clinical competence and social accountability [27,28].

### Quality of care assessment and impact evaluation

To ensure that SLC outputs translate into measurable impact rather than activity metrics alone, the HI model employs a triangulated evaluation framework. Qualitative data are gathered through focus group discussions with community members, yielding insights into patient satisfaction, perceived service quality, and barriers to engagement that quantitative data alone cannot capture [29,30]. Quantitative impact is assessed through structured numerical analysis of diagnostic outputs, medication dispensation, and referral completion rates, mirroring established approaches in health impact research [31,32]. Referral tracking, a dimension absent from many SRFC models in the literature, provides a concrete downstream metric of clinical effectiveness by confirming whether patients with urgent conditions receive the required specialised care.

### Strengths, limitations, and future directions

The primary strength of this study is its detailed, transparent documentation of an operational healthcare delivery model in a resource-constrained LMIC setting. The structured patient flow protocol, tiered consultation system, standardised data collection framework, and NGO-supervised referral pathway represent a degree of operational rigour which is uncommon among student-led community health initiatives, particularly in Pakistan. We purposefully designed this level of documentation to function as a replicable blueprint for other student organisations and medical institutions seeking to implement similar models.

Several limitations must be acknowledged. We report data from only two clinic cycles. The absence of longitudinal follow-up data limits the ability to quantify chronic disease outcomes, referral completion rates, and changes in health literacy. Patient satisfaction instruments, validated outcome measures, and structured student learning assessments were not implemented during these early cycles, limiting the ability to evaluate broader impact. The small sample size and single-site design preclude generalisability to the broader Pakistani population. However, similar structural and socioeconomic conditions are shared by hundreds of communities across Pakistan’s major cities, many of which remain effectively isolated from both the formal public and private healthcare sectors. Our findings therefore suggest that the HI model may be replicable across similar settings, although this remains to be formally demonstrated. Potential investigator bias must also be acknowledged, given that the implementing organisation conducted the study. The absence of external evaluation is an additional limitation that should be addressed in subsequent phases of the initiative.

### Strategic recommendations

The evidence presented here suggests several strategic priorities for those seeking to replicate or scale the SLC model in similar settings. The immediate priority for the HI initiative is strengthening the data infrastructure, studying standardised clinical outcome indicators and formal educational assessment to generate evidence needed to attract institutional partnerships and sustainable funding. Longitudinal tracking of patients with chronic conditions, even through brief telephone follow-up between SLC cycles, would substantially strengthen the model’s ability to demonstrate impact on chronic disease management outcomes.

The HI experience offers several lessons for similar student organisations considering adoption of the SLC model. Community partnership should be viewed not as a peripheral logistical consideration but as a foundation for the clinic’s legitimacy and uptake. A structured volunteer competency framework is also essential to maintaining care quality and patient safety. Additionally, sustainability requires diversified funding through institutional partnerships, government engagement, and structured philanthropy rather than reliance on crowdfunding alone. Sharing operational blueprints through academic publications, conference presentations, and inter-university training workshops will be important for supporting replication across Pakistan’s diverse medical education landscape.

Finally, integrating telemedicine and digital health tools could extend SLC services to peri-urban and rural communities where physical deployment is constrained by distance and logistical barriers. Digital health initiatives in comparable LMIC settings have demonstrated feasibility and community acceptability, suggesting that integrating such tools into the SLC workflow could expand the model’s geographic reach while limiting additional operational cost [33–35].

## CONCLUSIONS

The SLC model demonstrates that community-embedded, student-facilitated primary healthcare is operationally feasible, affordable, and potentially clinically meaningful in one of Pakistan’s underserved urban communities. With a direct medical care cost of under USD 0.50 per patient, the model offers a financially accessible and potentially replicable approach to addressing gaps in primary healthcare access. By deploying supervised medical students in task-sharing roles, integrating health education into routine encounters, and establishing referral pathways where none previously existed, the SLC model addresses key dimensions of Universal Health Coverage, including financial risk protection, equitable access to essential services, and quality of care. It also provides medical students with early, supervised exposure to the social determinants of health and the practical challenges of delivering care at the margins of formal health systems – experiences that cannot be fully replicated in classroom or hospital settings. With rigorous evaluation, sustained funding, and coordinated scale-up, the SLC model could contribute to reducing healthcare inequalities in Pakistan.

## Data Availability

**Data availability:** No formal data sets were generated or analysed during the current study. All information presented is derived from descriptive observations recorded during humanitarian field activities. Summarised data are available from the corresponding author upon reasonable request. Any requests regarding details about HI’s operations for replicating the SLC model can be made *via* email to the corresponding author.
